# GeneMiner2: Accurate and Automated Recovery of Genes From Genome Skimming Data

**DOI:** 10.1111/1755-0998.70111

**Published:** 2026-02-18

**Authors:** Xinyi Yu, Zizhen Tang, Zhen Zhang, Yongxiu Song, Hao He, Yi Shi, Jiaqing Hou, Yan Yu

**Affiliations:** ^1^ Key Laboratory of Bio‐Resources and Eco‐Environment of Ministry of Education, Southwest Bio‐Resources R&D Key Laboratory of Sichuan Province, Laboratory for Ex Situ Conservation and Resource Utilization of Montane Plants, College of Life Sciences Sichuan University Chengdu Sichuan China; ^2^ Chengdu Botanical Garden Chengdu Sichuan China

**Keywords:** adaptive *k*‐mer selection, assembly tool, fine‐grained read selection, phylogenetic marker

## Abstract

With the growing accessibility of low cost genome skimming, large‐scale recovery of target genes in non‐model species has become feasible, providing strong data for phylogenomic and evolutionary studies. However, existing tools for data assembly including Read2Tree, HybPiper, and GeneMiner still suffer from computational difficulty and assembly errors when processing genome skimming data, especially for low‐level taxa that lack closely related genome references. Here, we present GeneMiner2, an updated version of GeneMiner, as an efficient and automated gene assembly and phylogenetic tool to overcome these limitations. Compared with the previous version, GeneMiner2 introduces three key optimizations by deploying a two‐level hash table that speeds up *k*‐mer filtering, applying fine‐grained read selection with strand‐orientation and structural‐anomaly detection to handle complex heterozygous regions, and incorporating adaptive *k*‐mer selection in the de Bruijn assembler. Together enabling accurate assembly of target genes without the need for closely related references. Using simulated datasets with low coverage and divergent references, we validated the robustness and improved accuracy of GeneMiner2 in the assembly of single‐copy genes. Moreover, using genome skimming data from the subfamily Apioideae (Apiaceae), GeneMiner2 outperformed Read2Tree and HybPiper in accuracy, completeness, and speed. Equipped with a user‐friendly graphical interface, GeneMiner2 integrates functions including assembly quality control, paralog detection, tree reconstruction, and divergence time calibration, offering a robust and efficient solution for phylogenetic inference using genomic level data. GeneMiner2 supports cross‐platform operation. GeneMiner2 provides a user‐friendly graphical interface for desktop users, and its high‐performance command‐line interface is specifically optimised for high‐throughput analyses on Linux servers and computing clusters. Installation instructions, detailed documentation, and source code are available on GitHub (https://github.com/sculab/GeneMiner2).

## 1 Introduction

The pace of sequencing and the quality of genome assemblies have risen significantly over the past two decades. Current technologies have permitted large‐scale genome sequencing and assembly for all species. Ambitious initiatives are underway to generate a comprehensive genomic reference for eukaryotic life. Although the Earth BioGenome (Lewin et al. 2018) and 10KP (One Thousand Plant Transcriptomes 2019) projects have generated high‐quality genome assemblies for many organisms to fill in taxonomic sampling gaps, numerous lineages still lack adequate genomic resources in public databases or have not yet had their genomes sequenced with high coverage. This data gap poses significant challenges for phylogenetic reconstruction based on genomic data, a foundation for studying evolutionary history and processes (e.g., hybridisation and adaptation) in non‐model organisms. Short read sequencing methods represented by genome skimming have rapidly reduced technical barriers and sequencing costs (Do et al. 2013), and are being widely applied to non‐model taxa to obtain large‐scale genomic data (Malukiewicz et al. 2021; Nawy 2011; Quattrini et al. 2024).

To date, publicly released genomic data cover 57,230 Eukaryota species, of which 20,710 constitute reference genomic data and 9850 are annotated. This imbalance leaves most phylogenetic lineages without high‐quality reference genomes, creating a significant bottleneck for genome assembly. Notably, current reference‐based methods rely heavily on close relatives and face significant limitations when applied to taxa divergent from available references. De novo genomic assemblies, while independent of related references, often fail to resolve alternative isoforms and duplicated genes when lacking high‐coverage sequencing libraries. Moreover, as data volumes expand, de novo approaches often fail to recover complete gene sequences, especially for low‐coverage or fragmented datasets.

The target‐enrichment methods have been an alternative for capturing sequences of specific loci or genes with improved sequence completeness and reliability. A common method to assemble the target gene sequences has been the mapping‐based approach (Johnson et al. 2016) (Allen et al. 2015; Dylus et al. 2024), which works by directly processing raw reads into groups corresponding to different target genes, a way bypassing the genome assembly. However, when there is significant genetic divergence between the reference sequence and the studied lineage, this approach can lead to issues such as initial mapping failure, assembly errors, and unreliable results. Although existing software has adopted strategies like gene‐specific de novo assembly (Johnson et al. 2016) and iterative assembly (Allen et al. 2015), errors remain unavoidable, particularly when applied to genome skimming data characterised by incomplete genomic coverage.

The volume of eukaryotic genome skimming sequencing datasets on NCBI has reached the scale of hundreds of thousands currently. Our laboratory developed Easy353 (Zhang et al. 2022) and GeneMiner (Xie, Guo, et al. 2024; Liu et al. 2024), reference‐guided gene assemblers that support whole genome sequencing (including genome skimming data), target enrichment sequencing, and transcriptome sequencing. Easy353 utilises an optimised filtering approach based on *k*‐mers and an assembly algorithm that employs the weighted de Bruijn graph. This method enhances the ability of identifying appropriate reference sequences and has been successfully applied to the extraction of Angiosperm353 genes from genome skimming data in a wide range of taxa from divergent plant families such as Papaveraceae (Chen et al. 2023) and Orchidaceae (Zhang et al. 2024). The method was also successfully used to assemble low‐copy genes of Arthropods (Wang et al. 2025). Building upon Easy353, GeneMiner optimises the weighted node model and integrates re‐filtering with a soft‐boundary mechanism to enhance assembly accuracy. This pipeline has successfully extracted single‐copy genes (SCGs) and Angiosperm353 genes from NGS datasets in diverse plant taxa, including Lamiaceae (Jin et al. 2025; Wang, Wu, et al. 2024), Bambusoideae (Wang, Li, et al. 2024), Nitrariaceae (Xu et al. 2025), Gesneriaceae (Xie, Peng, et al. 2024), and Liliaceae (Cheng et al. 2025). Although Easy353 and GeneMiner demonstrate high speed, gene recovery rate, and accuracy, the assembly reliability remains limited success with very divergent reference sequences, especially for genome skimming data.

The GeneMiner2 we present in this article is designed to rapidly assemble target genes in the absence of comprehensive references. Compared to the previous version, GeneMiner2 adopts an efficient two‐level hash table for read filtering, achieving a significant increase in processing speed while improving filtering accuracy. Fine‐grained read selection, incorporating strand orientation and structural anomaly detection, is introduced to address challenges associated with complex genomic regions and heterozygous structures. In the assembly module, GeneMiner2 implements a gene‐specific adaptive *k*‐mer selection strategy, making de Bruijn graph‐based assembly more suitable for genome skimming data. To improve downstream accuracy and efficiency, the pipeline integrates key modules for phylogenomic analysis and incorporates functions such as homology detection, reference‐based trimming, and divergence filtering to identify mis‐assemblies and non‐orthologous sequences. Additionally, GeneMiner2 offers robust cross‐platform compatibility, supporting Windows, Mac and Linux operating systems, and provides a user‐friendly GUI interface for Windows and Mac users.

## 2 Materials and Methods

### 2.1 Description of GeneMiner2

**FIGURE 1:**
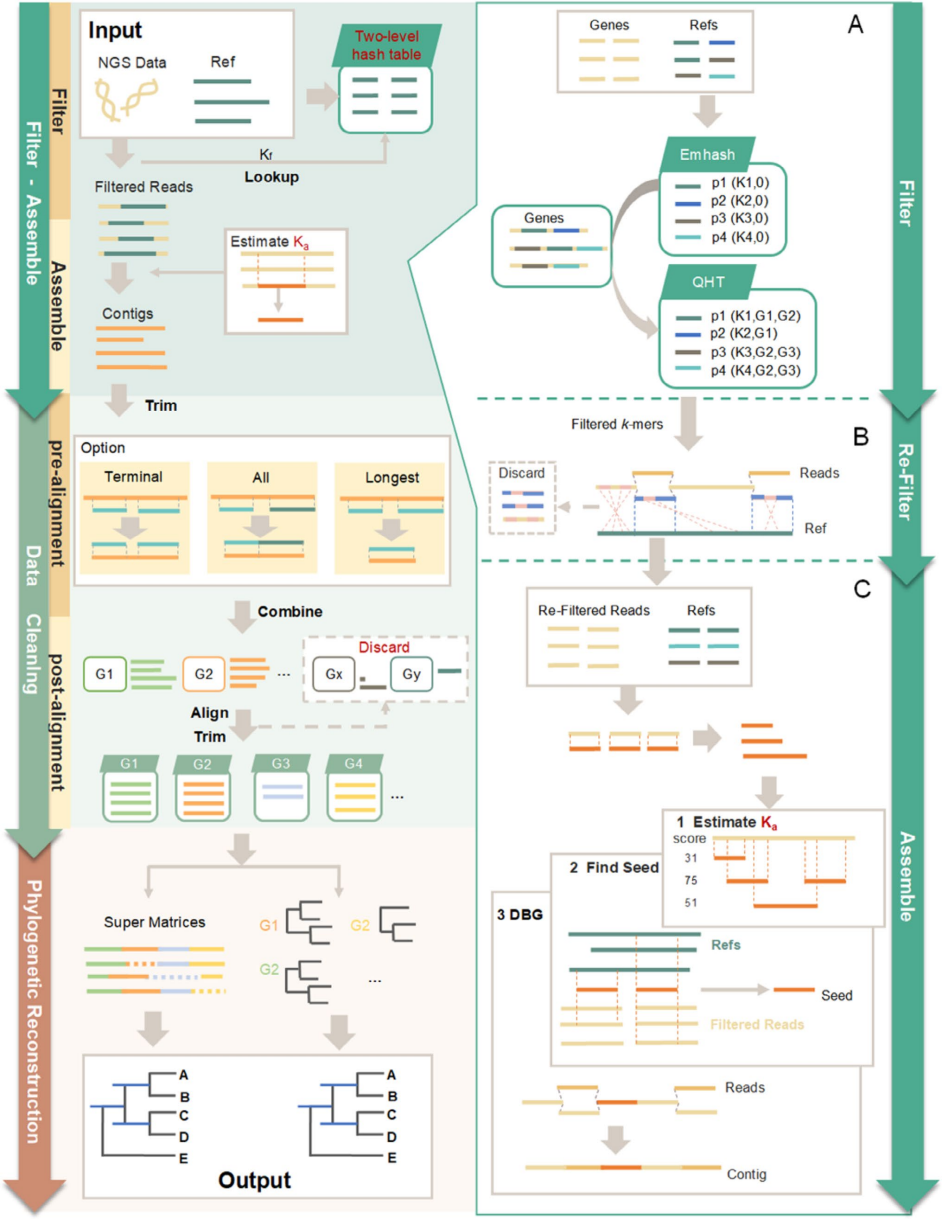
Overview of the GeneMiner2 workflow. GeneMiner2 pipeline from raw reads to phylogenetic tree (left panel). Three key optimisations were introduced (right panel): (A) a two‐level hash table for efficient *k*‐mer filtering, (B) strand orientation and structural anomaly detection for read selection, and (C) adaptive *k*‐mer selection in the de Bruijn graph assembler.

**FIGURE 2:**
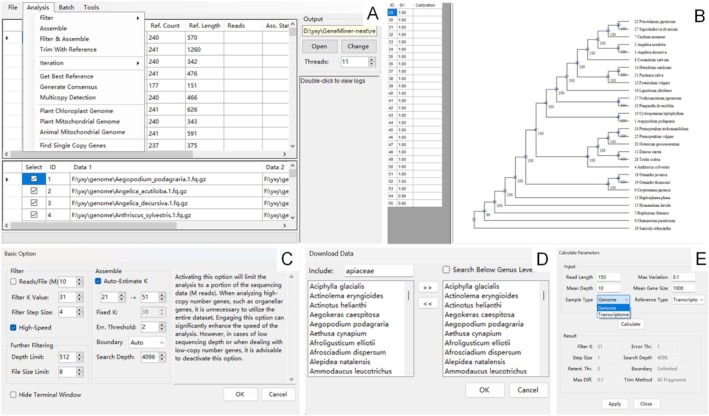
(A) The interface of GeneMiner2‐GUI. (B) An example of a resulting phylogenetic tree reconstructed from assembled genes, with bootstrap support values displayed at each node. (C) Basic options for filtering and assembly. (D) A built‐in online database was constructed via the NCBI and is used for reference retrieval. Users can filter by family or genus, and select species of interest for analysis. (E) Automated parameter calculator for different input types.

GeneMiner2 implements a streamlined workflow from raw sequencing reads to a time‐calibrated phylogeny (Figure 1). Starting with genome skimming or transcriptome reads together with reference sequences retrieved from its built‐in database, the pipeline applies a two‐level hash‐table *k*‐mer index for filtering, then performs fine‐grained read selection to discard reads that carry strand inversions or other structural anomalies. It next estimates an optimal *k*‐mer size ($k_{a}$) and assembles the filtered reads into contigs with a reference‐guided de Bruijn‐graph method. GeneMiner2 aligns contigs to their references, discarding paralogues that fail homology or coverage checks. Its *Calculate Parameter* tool (Figure 2E) reads sample statistics—read length, depth, data type and mean gene length—and returns length and divergence thresholds for BLAST trimming and downstream MSAs (MAFFT 7.52 [Katoh et al. 2002], MUSCLE 5.1 [Edgar 2022] and trimAL v1.2 [Capella‐Gutierrez et al. 2009]). A phylogenetic tree (FastTree 2.1 [Price et al. 2010], IQ‐TREE v3.0.1 [Nguyen et al. 2015], ASTRAL‐III [Zhang et al. 2018]) is inferred based on the alignments, and divergence times are then calibrated on the resulting topology (MCMCTree [Rannala and Yang 2007; Yang et al. 2006]).

### 2.2 Dataset Preparation

To systematically evaluate the performance of GeneMiner2 in recovering target genes and conducting phylogenetic analyses at low‐level taxa, we utilised an Illumina‐based sequencing dataset comprising genomic DNA (3–10×) and high‐depth RNA‐seq (6–7 G) data from 27 representative species of Apioideae (Apiaceae), covering lineages from basal to derived positions within the subfamily (Banasiak et al. 2013). For reference datasets, we retrieved the Angiosperms353 gene sets for 240 Apiaceae species using a built‐in online reference database. Acknowledging that the scarcity of high‐quality reference genes or well‐annotated genomes often restricts the accuracy of molecular marker extraction, we integrated OrthoFinder (Emms and Kelly 2019) into GeneMiner2. This process inferred and supplemented 2605 putative single‐copy genes (SCGs) from the transcriptomes of closely related species, which were then constructed into an auxiliary reference sequence set.

### 2.3 Fast Read Filtering

GeneMiner2 extracts a set of relevant reads from raw sequencing data before further analysis. In this step, all reference *k*‐mers are stored in a database (Figure 1A). Reads will be assigned to one or more genes based on their constituent *k*‐mers. Therefore, targeted gene assembly is accelerated by operating on a reduced set of reads.

A nucleotide sequence is treated as a string over the alphabet $\left\{A,C,G,T\right\}$. Given a sufficiently long reference sequence, a reference *k*‐mer $K_{i}$ is defined as the i‐th substring of length k from the reference sequence R, namely $K_{i}=R_{j}\left(i\leq j<i+k\right)$. The *k*‐mers of a reference sequence form a sequential list $\left(K_{1},K_{2},\cdots ,K_{\left|R\right|-k+1}\right)$. Similarly, the *k*‐mers of a read also form a sequential list of all its *k*‐substrings.

To allow efficient query with a gene's constituent *k*‐mers, the *k*‐mer database must be able to perform three tasks:

1. Given a *k*‐mer K and a gene g, insert a key‐value pair $\left(K,g\right)$ in amortised $O\left(1\right)$ time.
2. Given a *k*‐mer K and a gene g, check the existence of key‐value pair $\left(K,g\right)$ in amortised $O\left(1\right)$ time.
3. Given a *k*‐mer K, find all unique key‐value pairs $G=\left\{\left(K,g_{1}\right),\left(K,g_{2}\right),\cdots \right\}$ in amortised $O\left(\left|G\right|\right)$ time.

These requirements closely resemble what is known as a *multimap*. A difference is that we require key‐value pair deduplication in addition to unique keys, which may not be efficient in all implementations. In the previous version of GeneMiner, the *k*‐mer database is built around Python's dict type, which incurs significant overhead and limits throughput. To improve performance, GeneMiner2 implemented a custom data structure for *k*‐mer lookup (Figure S12), which is shown to be at most 960% faster than the previous version (Figure S13).

A two‐level hash table is designed as the basis of *k*‐mer database, where Level 1 stores *k*‐mers and Level 2 tightly packs sets of integers that represent genes. At Level 1, a generic hash table is used to map each *k*‐mer to a pointer into its corresponding region at Level 2. Each such region at Level 2 can be interpreted as a special type of hash table, containing a set of integers. Hence, we achieve efficient value deduplication without the overhead of maintaining auxiliary data structures.

In terms of implementation, Level 1 uses *emhash* (https://github.com/ktprime/emhash) as the underlying storage. When inserting the relationship between a *k*‐mer K and a gene g, we simply encode K and stored it at Level 1 with an empty value as key‐value pair $\left(K,0\right)$. We then store the presence of g in Level 2 and obtain a pointer to the corresponding region. The value in key‐value pair $\left(K,0\right)$ is then populated with the pointer. As Level 2 updates, pointers at Level 1 are continually fixed up. While slightly slowing down insertion, this allows fast *k*‐mer queries because pointers at Level 1 always point to the correct region. In addition, *emhash* is able to reach a high load factor (>0.9) without much performance loss, significantly reducing memory consumption.

Level 2 is implemented as a collection of Quotient Hash Tables (QHT) (Géraud et al. 2019) with 2 buckets per row, where each QHT holds a set of integers representing all genes that contain a specific *k*‐mer. Different from the original QHT, our implementation does not store elements as *fingerprints*, but instead achieves compactness via the ‘simple’ variant of Compact Hashing via Bucketing (Köppl et al. 2022). In other words, we group rows into $2^{h}$ blocks; for each gene g, we store a *quotient* $f\left(g\right)\;\mathrm{mod}\;2^{h}$ in the $\left\lfloor f\left(g\right)/2^{h}\right\rfloor$‐th block, where $f\left(x\right)$ is the hash function and h controls the quotient size. We also implemented partial‐key cuckoo hashing, allowing an element to be stored in two rows in the same block instead of one, in a way that resembles Cuckoo Filter (Fan et al. 2014). Although this change creates overhead by increasing associativity, it saves memory by increasing load factor. Furthermore, identical QHTs inside Level 2 are deduplicated to save memory. As a result, the implementation is very memory‐efficient even when written in a garbage‐collected language. Overall, we enjoy amortised $O\left(1\right)$ time complexities with vastly improved memory consumption, by employing separate memory‐efficient components in both levels.

QHT is designed to be probabilistic, but in the context of *k*‐mer matching, it is reasonable to consider our variant deterministic. Because we do not use fingerprints to represent elements, false positives are eliminated. The probability of false negatives is tightly linked to the frequency of insertion failure. When a *k*‐mer is shared by too many genes, it can be simply ignored as it provides no information in *k*‐mer filtering, so we assume the number of genes n to be bounded in each QHT. If we allow insertion to fail at most once, we derive the failure probability ϵ≤1/n, which results in a worst‐case lower bound $l+s=\Omega \left(\log n/\log\log n\right)$ (Yeo 2023), where l is the count of buckets per row and s is the count of overflow buckets. By choosing n=1024 as a generous bound, we arrive at l=2 and s=2, which produce no false negative on a wide range of real‐world genomes. Even in the rare case of insertion failure, dropping a small number (<10) of key‐value pairs will not affect the validity of *k*‐mer matching because a gene has hundreds of *k*‐mers. In conclusion, our construction can provide fast, reliable *k*‐mer matching with virtually no false negatives.

### 2.4 Fine‐Grained Read Selection

While the previous version of GeneMiner has employed an iterative filtering approach to refine the reads, the removal of erroneous reads is somewhat capricious. Some types of erroneous reads significantly degrade the assembly results because they complicate the de Bruijn graph in an unexpected way. For example, multiple displacement amplification generates inverted chimeras with high abundance (Lu et al. 2023), causing elevated error rates in single‐cell sequencing data. Due to the use of a weighted de Bruijn graph, GeneMiner is especially sensitive to inversions in sequencing data: if an inversion in sequencing reads happens to skip over a variable region, it might receive a high weight due to its apparent *k*‐mer similarity to the reference, despite having a structural difference (Figure S14). Additionally, even if a structural variation is resolved without error, few ways exist to leverage its phylogenetic information. Consequently, we incorporated statistical testing as well as improved heuristics into iterative read filtering to alleviate the interference of large structural variation (Figure 1B).

#### 2.4.1 Runs Test

When a read contains multiple inversions in relation to the reference, it becomes effectively unalignable and its location relative to the reference cannot be efficiently determined. It also tangles the de Bruijn graph by creating loops, causing unintended contig breaks. We remove such reads with a Wald–Wolfowitz runs test.

In this test, we iterate over the *k*‐mers of the read. For each *k*‐mer $K_{i}$, we calculate its reverse complement ${\bar{K}}_{i}$ and test whether $\left(K_{i},g\right)$ or $\left({\bar{K}}_{i},g\right)$ exists in the reference database. If $\left(K_{i},g\right)$ exists but $\left({\bar{K}}_{i},g\right)$ does not, the *k*‐mer is matching in the forward direction. Similarly, if $\left({\bar{K}}_{i},g\right)$ exists but $\left(K_{i},g\right)$ does not, the match is in the reverse direction. The other two cases are ignored. The forward and reverse directionalities form a sequence, which can be tested statistically. Let the total number of forward matches be $n_{1}$ and reverse matches $n_{2}$. We calculate the expected number of runs and the variance as follows.

$$E\left(R\right)=\frac{2n_{1}n_{2}}{n_{1}+n_{2}}+1$$

$$\sigma^{2}=\frac{2n_{1}n_{2}\left(2n_{1}n_{2}-n_{1}-n_{2}\right)}{{\left(n_{1}+n_{2}\right)}^{2}\left(n_{1}+n_{2}-1\right)}$$

As most reads unambiguously match the reference in only one direction, we expect the directionality of *k*‐mer overlaps to show strong positive autocorrelation. We require the test statistic $z=\frac{r-E\left(R\right)}{\sigma }$ to be in the lower tail with at least $10^{-5}$ significance to accept a read. Although removing some reads may introduce bias and information loss, we argue that these reads contain little usable information for phylogenetics. They generate unreliable alignments and deteriorate the accuracy of phylogenetic inference. When included, they cause bias on their own rather than reduce the overall inference bias. Hence, we opt to use only reads without frequent inversions to ensure stable extraction of phylogenetic information.

#### 2.4.2 Longest Run Test

This test is a crude approximation of the well‐known test for multiple hits on the same diagonal of a comparison matrix (Wilbur and Lipman 1983). We ignore the distance between hits and inspect only the longest run in each direction, which massively speeds up the test. The null hypothesis is that matching *k*‐mers have random directions with probability 0.5. While the random hypothesis is already rejected using the runs test, we have to note that the longest run test focuses on local *k*‐mer chains rather than global randomness. In this regard, we do not use a conditional statistic because this test has little equivalence to the runs test and is not a refinement.

A very accurate estimator of the longest run is given by Flajolet and Sedgewick (Flajolet and Sedgewick 2009), where n is the sample size, γ the Euler–Mascheroni constant and $P\left(x\right)$ an oscillating function with small fluctuations ($<10^{-6}$).

$$\begin{array}{c} E_{n}\left(L\right)=\log_{2}n+\frac{\gamma }{\ln2}-\frac{3}{2}+P\left(\log_{2}n\right)\\ +O\left(\frac{{\left(\ln n\right)}^{2}}{\sqrt{n}}\right)\approx \log_{2}n-0.6676 \end{array}$$

It is known that L follows a Gumbel distribution asymptotically. Considering each run as an independent sample from a geometric distribution, we obtain a lower bound of $P\left(L\leq E_{n}\left(L\right)+m\right)$ by substituting it with the probability that all $E\left(R\right)$ expected runs are shorter than $E_{n}\left(L\right)+m$. With m=4, we also show that the probability is monotonically increasing over n.

$$\begin{array}{c} P\left(L\leq E_{n}\left(L\right)+4\right)\approx {\left(1-0.5^{E_{n}\left(L\right)+5}\right)}^{E\left(R\right)}\\ ={\left(1-0.5^{\log_{2}n+\frac{\gamma }{\ln2}+3.5}\right)}^{\frac{n}{2}+1}\approx {\left(1-\frac{0.0496}{n}\right)}^{\frac{n}{2}+1} \end{array}$$

$$\begin{array}{c} \frac{d}{\mathrm{dn}}P\left(L\leq E_{n}\left(L\right)+4\right)\\ \approx \frac{{\left(\sqrt{\frac{n-0.0496}{n}}\right)}^{n}\left(0.0496n+\left(n-0.0496\right)n\ln\left(\frac{n-0.0496}{n}\right)+0.0992\right)}{2n^{2}} \end{array}$$

when n=2, $P\left(L\leq E_{n}\left(L\right)+4\right)\approx 0.9510>0.95$, so the inequality holds for every n≥2. Therefore, under the null hypothesis, the longest run generally satisfies $L\leq E_{n}\left(L\right)+4$ with 95% confidence. Indeed, the results by Makri and Psillakis (Makri et al. 2011) supports that the expected occurrences of runs whose length satisfy $L>E_{n}\left(L\right)+4$ are numerically less than 5%. This condition allows us to reject the null hypothesis with 95% significance in a one‐tailed test. We also show that this test is not redundant: as $E_{n}\left(L\right)+4>\frac{n}{E\left(R\right)-3.74\sigma }$ holds for n≥19, reads that pass the runs test do not necessarily have long *k*‐mer runs. Consequently, we can remove reads without long same‐direction *k*‐mer runs and assign directions to the remaining reads depending on the direction of their longest run. If a read has long runs from both directions, we employ a third test to determine the validity of the read.

#### 2.4.3 *k*‐Mer Identity Test

If a read has long runs of *k*‐mers in both directions, its orientation is considered ambiguous, but in many cases the read is perfectly valid. For example, small transposons in the reference may easily become inverted in other species, which should not be excluded from gene phylogeny. We use the percentage identity of *k*‐mers as a heuristic to decide which region to trust. If the longest run in one direction has significantly more gaps than the other direction, it is possible that only one of the runs is actually homologous to the reference. In contrast, if two runs from opposite directions are equally similar to the reference, we have no means to resolve the ambiguity and can only remove the read.

For reads that have a forward region with $n_{1}$ *k*‐mers in total and a reverse region with $n_{2}$ *k*‐mers, let their proportions of *k*‐mers matching the reference be $p_{1}$ and $p_{2}$ respectively, and we can test the null hypothesis $p_{1}=p_{2}$ with a *z*‐score following the standard normal distribution.

$$z=\frac{p_{1}-p_{2}}{\sqrt{\frac{p_{1}\left(1-p_{1}\right)}{n_{1}}+\frac{p_{2}\left(1-p_{2}\right)}{n_{2}}}}$$

If the *z*‐score does not reject the null hypothesis with 95% significance in a two‐tailed test, the read is removed. Otherwise, the read is kept because it might have spanned an element with a different history from its flanks. The assembler would be able to differentiate its orientation based on the uneven similarity to the reference. As a result, we can make use of information in most reads without risking misassembly.

A summary of the three tests is given in Figure S15. It should be noted that the statistical tests mentioned above require small *k*‐mer sizes. Because most reads have no orientation conflicts, less than 1% of total reads are removed in this step. This allows us to improve the assembly of certain difficult genes without sacrificing accuracy. In addition, we implemented an improved strategy for read filtering. We remove discordant read pairs eagerly using the conclusions drawn from statistical tests. If the coverage drops below a threshold during iterative filtering, we revert the last iteration and stop filtering. This prevents overzealous read removal in low coverage regions, resulting in higher contiguity and thus better sequence recovery.

### 2.5 Adaptive *k*‐Mer Selection

GeneMiner2 assembles individual genes by walking on the de Bruijn graph (Idury and Waterman 1995). This approach is known to be sensitive to the selection of *k*‐mer size (Schatz et al. 2010). Hence, in order to retrieve high‐quality sequences, it is of paramount importance to choose the optimal *k*‐mer size (Figure 1C).

Anchor *k*‐mers are defined as *k*‐mers that occur once in a read and overlap with the reference; unique anchor *k*‐mers refer to the first *k*‐mers in each consecutive run of anchors.

Intuitively, the more unique anchor *k*‐mers, the more chance to align a read correctly onto the reference. However, the possibility of misassembly also grows with the number of unique anchor *k*‐mers. Reducing k increases the number of anchor *k*‐mers, but also increases coincidental *k*‐mer overlaps. Additionally, larger values of k are generally desirable to improve assembly in repetitive regions. Unfortunately, it is not possible to obtain the best value of analytically, because a large *k*‐mer not only reduces spurious hits, but also helps in differentiating paralogs, whose statistical properties are largely unknown. Hence, we assign a score to *k* values heuristically to estimate the optimal value of *k*.

The strategy is to maximise k until the score falls below a threshold. Firstly, a limit on the length of consecutive anchor *k*‐mers is set. If a run of anchor *k*‐mers is long, more than one unique *k*‐mers will be counted, allowing it to win over coincidental overlaps. Secondly, instead of directly using the number of unique anchor k‐mers N, we define the score as

$$s=\frac{\mathrm{kN}}{l_{r}-k+1}$$

where $l_{r}$ is the length of the read. The score can be understood as normalised anchored bases: kN is the number of bases covered by unique anchor *k*‐mers and $l_{r}-k+1$ the number of *k*‐mers in the read. As the read gets longer, the expected number of anchors is also higher, so the number of anchored bases is normalised by the total number of *k*‐mers, reducing the bias favouring small *k*‐mers on relatively short reads. Lastly, we find the maximum k such that s is higher than half its maximum value. This is because the weighted de Bruijn graph assembler can work reasonably well when there are few *k*‐mer overlaps between the reads and the reference. While we do not want to lose most of the anchors, we consider it acceptable to reject half of anchored bases. Therefore, shorter anchor *k*‐mers are sacrificed to the benefits of a larger k, which make assembly more accurate. This strategy both lowers the error rate and improves the contiguity, enabling reliable phylogenetic inference.

### 2.6 Parameters Calculation and Settings

Genome assemblers are among the most user‐unfriendly pieces of software in bioinformatics. Determining the appropriate parameters is generally an arduous task because of the complex interaction between parameters (Rádai et al. 2024).

We anticipate that most users will not need to adjust the parameters of GeneMiner2, as the default configuration recommended by the parameter estimation module already prioritises accuracy. In this study, the assembly module was run with the parameter settings recommended by the ‘*Calculate Parameter*’ module across all datasets.

The module calculates several parameters from four arguments: the variation between samples (v), the average length of genes ($l_{g}$), the average sequencing depth (d) and the read length ($l_{r}$). We expect these arguments to be relatively easy to understand: only v necessitates an educated guess based on average nucleotide identity. The other three arguments can be inferred from the properties of the samples, easing the process of software configuration.

The *k*‐mer size in read filtering ($k_{f}$) is calculated based on the read acquisition rate $p=1-{\left(1-{\left(1-v\right)}^{k_{f}}\right)}^{l_{r}-k_{f}+1}$ using the formula proposed in the previous version of GeneMiner (Xie, Guo, et al. 2024). The tool tries twice to find the largest $k_{f}$: first using p≥0.99 as the constraint; p≥0.95 if the former is not possible. The lower limit is $k_{f}\geq 17$ as per the original recommendation. Otherwise, an error will be generated.

BLAST (Altschul et al. 1990) was employed to trim low‐quality fragments from assembled results. GeneMiner2 selects an appropriate alignment strategy based on the reference and sequencing data source, and removes sequences exceeding the *Retention Length Threshold*. Subsequently, GeneMiner2 applies a divergence filtering module that calculates the maximum pairwise divergence among sequences during multiple sequence alignment (MSA). To eliminate paralogs, sequences with pairwise divergence exceeding the *Maximum Difference* are excluded.

### 2.7 Benchmarking on Simulated Data

To evaluate the performance of GeneMiner2, we established a comparative framework that ran two independent analysis pipelines in parallel: the conventional assembly and annotation approach and the GeneMiner2 workflow. Simultaneously, the assembly accuracy and coverage of GeneMiner2 were assessed against the previous version.

**FIGURE 3:**
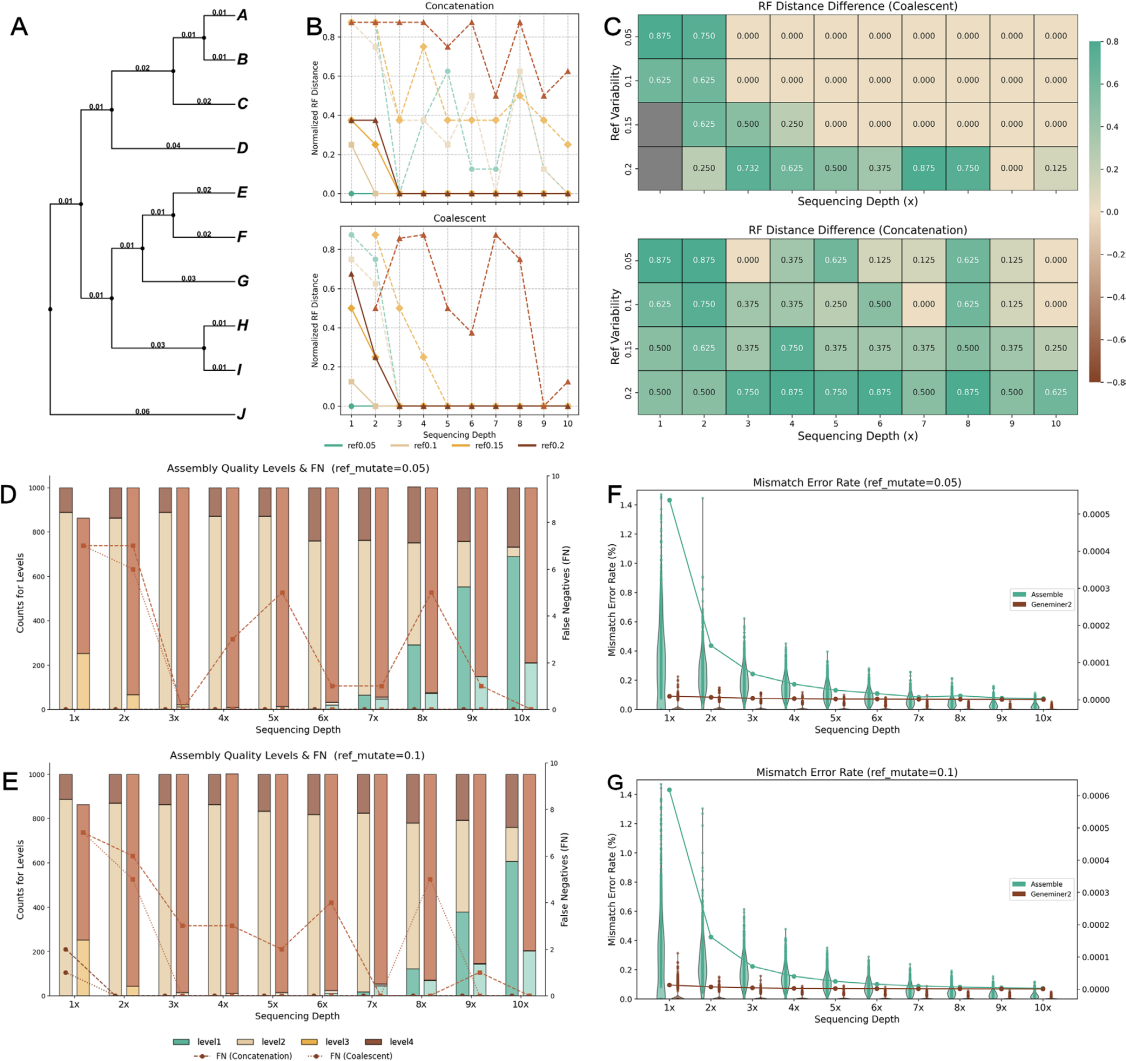
Performance of GeneMiner2 and the conventional approach under low‐coverage sequencing and divergent references. (A) The topology and branch lengths of the standard tree used for simulating sequencing data. (B) Normalised Robinson–Foulds (RF) distance differences between phylogenetic trees reconstructed by GeneMiner2 and the conventional approach, relative to the standard tree, across different sequencing depths and reference divergence levels. (C) The heatmaps display differences in RF distances of coalescent and concatenation‐based trees generated by the conventional approach versus GeneMiner2, relative to the standard tree. Green indicates that GeneMiner2 reconstructed trees closer to the standard tree, brown indicates greater accuracy of the conventional method, and grey marks cases where the species tree could not be recovered. (D) Quality of assembled sequences at a reference divergence of 0.05, comparing GeneMiner2 (left, dark bars) and the conventional approach (right, light bars). Green, beige, yellow and brown represent assembled sequences of high quality (L1), intermediate quality (L2, L3) and low quality (L4), respectively. Dashed and dotted lines indicate false negatives (FN) in coalescent and concatenation‐based trees, respectively, compared to the gold‐standard tree. (E) Quality of assembled sequences at a reference divergence of 0.1. (F) Mismatch error rates (%) compared to standard sequences at divergence 0.05. The violin plot and scatter plot illustrate the mismatch error rate for individual gene sequences, while the line chart shows the overall mismatch error rate as a proportion of the total recovered sequence length. Rates exceeding 1.5% were not displayed. (G) Mismatch error rates (%) compared to standard sequences at divergence 0.1.

The simulation framework included a ten‐taxon species tree (Figure 3A), with A–I assigned as the ingroup and J as the outgroup, and branch lengths were assigned with unequal values. Based on this topology, 1000 single‐copy genes were generated as standard sequences using Seq‐Gen v1.3.4 (Rambaut and Grass 1997). Each gene comprised ten 1000‐bp nucleotide sequences simulated under equal base frequencies (0.25) using the GTR model. Based on these sequences, we simulated paired‐end sequencing reads at varying coverages (1× to 10×; 150 bp, Q20) using NGSNGS v0.9.0 (Henriksen et al. 2023). To simulate reference divergence, mutations were introduced into the gold standard sequences at divergence levels of 0.05 to 0.2, enabling evaluation of GeneMiner2's performance in recovering target genes and reconstructing phylogenies using reference sequences with varying phylogenetic relatedness.

For the conventional approach, raw sequencing reads were first subjected to de novo assembly using MEGAHIT (Li et al. 2015), employing 20 threads (−t 20). To extract target gene sequences, a local nucleotide BLAST database was constructed from the reference gene set using makeblastdb. The assembled contigs were then mapped against this database using BLASTn (Altschul et al. 1990). The alignment results were generated in a customised tab‐separated format (−outfmt 6), capturing the query ID, subject ID, and alignment coordinates (qstart, qend). The recorded alignment coordinates were then used by a Python script to extract the corresponding target gene sequences from the contigs.

For the GeneMiner2 workflow, the assembly and sequence cleaning modules were run across all datasets using the parameter settings recommended by the ‘*Calculate Parameter*’ module. GeneMiner was run using the default, recommended parameters for sequence assembly.

To ensure the comparability between pipelines, we applied a unified sequence alignment and trimming strategy to the target sequences generated by both methods (MAFFT 7.52 [Katoh et al. 2002] and trimAL v1.2 [Capella‐Gutierrez et al. 2009]).

For phylogenetic reconstruction, all pipelines utilised identical parameters and methodologies to ensure a robust comparative analysis. The trees were inferred using two strategies, the coalescent method and the concatenation method, both of which were applied to the test datasets. For the coalescent method, single‐gene trees were constructed from the individual gene alignment datasets using FastTree 2.1 (Price et al. 2010) under the GTR + G model, and branch support was assessed using 1000 bootstrap replicates. The species tree was constructed using ASTRAL‐III (Zhang et al. 2018). For the concatenation method, multiple gene sequences from the test datasets were concatenated into a supermatrix, from which the final phylogenetic tree was inferred using FastTree 2.1 (Price et al. 2010) under the GTR + G model, also employing 1000 bootstrap replicates for branch support estimation. To evaluate sequence reconstruction accuracy, the assembled sequences were assessed in terms of identity, coverage, number of sequences recovered per gene, and the presence of indel or substitution errors. Based on these metrics, sequences were categorised into four quality levels: Level 1 (identity = 100%, coverage = 50%–100%, count = 7–10; high quality), Level 2 (identity = 100%, coverage = 0%–50%, count = 7–10; intermediate quality), Level 3 (identity = 100%, count = 1–6; intermediate quality), and Level 4 (containing indel or substitution errors; low quality).

Phylogenetic reconstruction performance was compared between pipelines by calculating Robinson–Foulds distances between the reconstructed species trees and the standard tree (Figure 3A,C). Under reference divergence levels of 0.05–0.1, false negative splits relative to the standard tree were used to assess topological differences (Figure 3D,E).

### 2.8 Performance Assessment on Empirical Data

To evaluate the performance of GeneMiner2 on empirical datasets, we conducted a comprehensive comparison with its predecessor (GeneMiner) and two state‐of‐the‐art pipelines, Read2Tree and HybPiper. The pipelines were assessed based on three primary metrics: the rate of sequence recovery and assembled length, the accuracy of phylogenetic reconstruction, and the runtime for assembly and phylogenetic reconstruction. For this comparative analysis, we processed identical DNA and RNA datasets with shared reference sets (except for Read2Tree) and applied both concatenation and coalescent phylogenetic methods. All downstream analyses employed the same tools for sequence alignment, trimming, and tree reconstruction.

We performed target gene assembly using both versions of GeneMiner, and the runtime was calculated on both Windows and Linux systems. GeneMiner2 was run with recommended default parameters. For GeneMiner, the minimum *k*‐mer frequency threshold (limit count) was set to automatic mode to eliminate potential low‐quality redundancy.

Read2Tree imposes strict formatting requirements on its reference database, including standardised gene identifiers, orthologous group (OG) IDs and accession number. Due to the difficulties encountered with Read2Tree's reference database construction and the limited species representation within the restricted reference database, we downloaded reference orthogroups for *Daucus carota* var. *sativa* and *Helianthus annuus* . Genome skimming and transcriptome datasets were processed independently with default parameter settings. In addition, to enable a direct comparison between the GeneMiner2 and Read2Tree pipelines, we also used GeneMiner2 to perform assembly and tree inference based on the same OMA reference set employed by Read2Tree.

For molecular marker extraction with HybPiper, we utilised the Angiosperms353 and single‐copy nuclear gene (SCG) reference sets generated by the GeneMiner2 pipeline and ran HybPiper with default parameters. However, when applied to genome skimming data, HybPiper recovered only a limited number of target genes, and the amount of data retained after paralog filtering was suboptimal. We therefore opted to use the assembled sequences directly for phylogenetic tree reconstruction.

For runtime benchmarking, all assembly and phylogenetic reconstruction analyses were executed on the same Linux system with an identical number of threads.

## 3 Results

GeneMiner2 is a versatile and efficient tool designed for assembling short gene fragments from genome skimming data. It improves upon GeneMiner by introducing redesigned algorithms to three core steps. A two‐level hash table is implemented for the filtering step (Figure 1A), enabling high‐throughput processing of short read sequencing data. The strategy for refining reads is improved using a combined algorithm, which runs statistical tests (run test, longest run test and *k*‐mer identity test) alongside iterative re‐filtering to detect misaligned structure (Figure 1B). This process reduces the incidence of misassembly caused by sample deterioration and sequencing bias. An algorithm for automatic *k*‐mer size estimation is also incorporated into the assembler (Figures 1C and 2C) to improve sequence contiguity and reduce error rate without manual intervention.

Building on these core advances, GeneMiner2 offers an automated pipeline from raw reads to phylogenetic inference (Figure 1). It integrates read filtering, target gene assembly, homology screening, alignment curation, phylogenetic tree construction, and divergence‐time estimation—all accessible through a user‐friendly graphical interface (Figure 2). By coupling the new algorithms with an integrated workflow, GeneMiner2 enables accurate and efficient phylogenomics.

### 3.1 Robust Performance Under Low‐Coverage and Divergent References

We assumed a ten‐taxon species tree with unequal branch lengths (Figure 3A) and simulated 1000 single‐copy gene sequences (standard sequences) based on this topology. From these, genome skimming datasets were simulated with coverage ranging from 1× to 10× and reference sequences with 0.05–0.2 divergence.

We assembled genes using GeneMiner and GeneMiner2, and conducted a parallel comparison with the conventional genome assembly and annotation approach. In the conventional approach, genome assembly was performed with MEGAHIT (Li et al. 2015), and gene annotation was carried out using BLAST (Altschul et al. 1990). For sequence reconstruction accuracy (Figure 3B), we evaluated correctness (identity), completeness (coverage), the sequence count per gene (counts), and error rate (including indels and substitutions). Reconstructed sequences were classified into four levels.

GeneMiner2 recovered the complete set of 1000 target sequences across 1× to 10× coverage levels. It robustly assembled high‐quality sequences under distantly related references, with more than 80% of assembled sequences classified as L1–L2. At 0.05 divergence, the number of accurately assembled sequences was 3‐ to 96‐fold higher compared to the conventional approach, and at 0.1 divergence, this ranged from 3‐ to 78‐fold. Assembled results generated by the conventional assembly and annotation approach under low coverage contained indels and substitutions, with the proportion of incorrectly assembled sequences (L4) exceeding 70%. As sequencing depth increased, the conventional approach gradually reduced the proportion of misassembled sequences, reaching around 20% at 10× coverage. By comparison, GeneMiner2 achieved over 80% correctly assembled sequences (L1–L3) even at 1×, with sequence completeness continuously improving as depth increased. At 10× coverage, over 60% of the assembled sequences exhibited high correctness and completeness.

The proportion of erroneous bases within mis‐assembled L4 sequences was further quantified (Figure 3F,G). Across the full coverage range of 1–10×, GeneMiner2 consistently maintained a low error rate, substantially lower than that of the conventional approach, and remained stable regardless of sequencing coverage. By contrast, the conventional approach accumulated substantial indel and substitution errors at 1–2× depth, with error rates gradually decreasing above 3×.

For phylogenetic reconstruction accuracy (Figure 3C), we compared the inferred trees against the standard tree (Figure 3A) under different divergence levels, quantifying both the number of conflicting splits (false negatives, FN; Figure 3D,E) and the normalised Robinson–Foulds (RF) distance. GeneMiner2 consistently reconstructed accurate and robust trees across all coverage levels. At a divergence level of 0.05, concatenated and coalescent‐based trees with correct topologies were consistently reconstructed across 1–10× sequencing depths. At divergence 0.1, correct topologies were also stably reconstructed at depths of 2× and above. The conventional approach exhibited substantial fluctuations in concatenation‐based inference, whereas accuracy under the coalescent framework gradually stabilised at depths above 3×. Further comparisons based on normalised RF distance (Figure 3C) demonstrated that GeneMiner2 generally outperformed the conventional approach in reconstructing accurate phylogenetic topologies, especially for concatenation‐based trees under low coverage (1–5×) and high reference divergence (0.2).

We conducted a comparative analysis of the sequences recovered by GeneMiner and GeneMiner2 (classified as L1 and L2), focusing on those with no assembly errors (identity = 100%) and high recovery (at least 70% of the sequences were successfully recovered in each gene file). We also assessed the phylogenetic accuracy of the assemblies by quantifying the number of false‐negative branches (FN) in the reconstructed trees. Under low‐coverage conditions, GeneMiner2 recovered more than eight‐fold more high‐quality sequences than the previous version, and across all coverage levels it consistently yielded a greater number of high‐quality assemblies (Table S3). The higher assembly accuracy of GeneMiner2 resulted in more reliable phylogenetic inferences: for both the concatenation‐based and coalescent‐based methods, GeneMiner2 produced fewer false‐negative branches (FN) than the previous version across 1–10× coverage. The FN values for both versions of GeneMiner were lower than those obtained with the conventional approach (Figure 3D,E; Tables S4 and S5).

### 3.2 GeneMiner2 Outperforms Existing Tools

Our phylogenetic and assembly analyses were based on two reference resources: the Angiosperms353 gene sets retrieved from a public database, and 2605 putative single‐copy genes (SCGs) which were inferred from the transcriptomes of closely related species through GeneMiner2's integrated OrthoFinder (Emms and Kelly 2019). With the exception of Read2Tree, which did not support the required reference sequence format, all other pipelines utilised the identical set of reference sequences for molecular marker extraction and subsequent phylogenetic reconstruction.

Based on the same reference set, we quantified the number of assembled gene files, the mean number of sequences per gene file, and the mean assembled sequence length for GeneMiner, GeneMiner2 and HybPiper. For both the Angiosperms353 and single‐copy gene sets, GeneMiner2 consistently achieved higher sequence recovery than the predecessor GeneMiner, whereas HybPiper recovered far fewer sequences—only about one quarter of those obtained by either version of GeneMiner (Table S1). GeneMiner and HybPiper produced longer assembled sequences than GeneMiner2. This difference reflects the new assembly strategy implemented in GeneMiner2, which can accurately reconstruct a larger number of gene sequences, including very short ones, while imposing stricter control over assembly quality than the previous version and comparable pipelines, thereby reducing misassembled regions. For Read2Tree, the reference sequence format was incompatible with our standard evaluation, so we instead quantified, for its intermediate file, the mean number of sequences per gene alignment file. Under this metric, GeneMiner2 still recovered the highest average number of sequences per gene.

**FIGURE 4:**
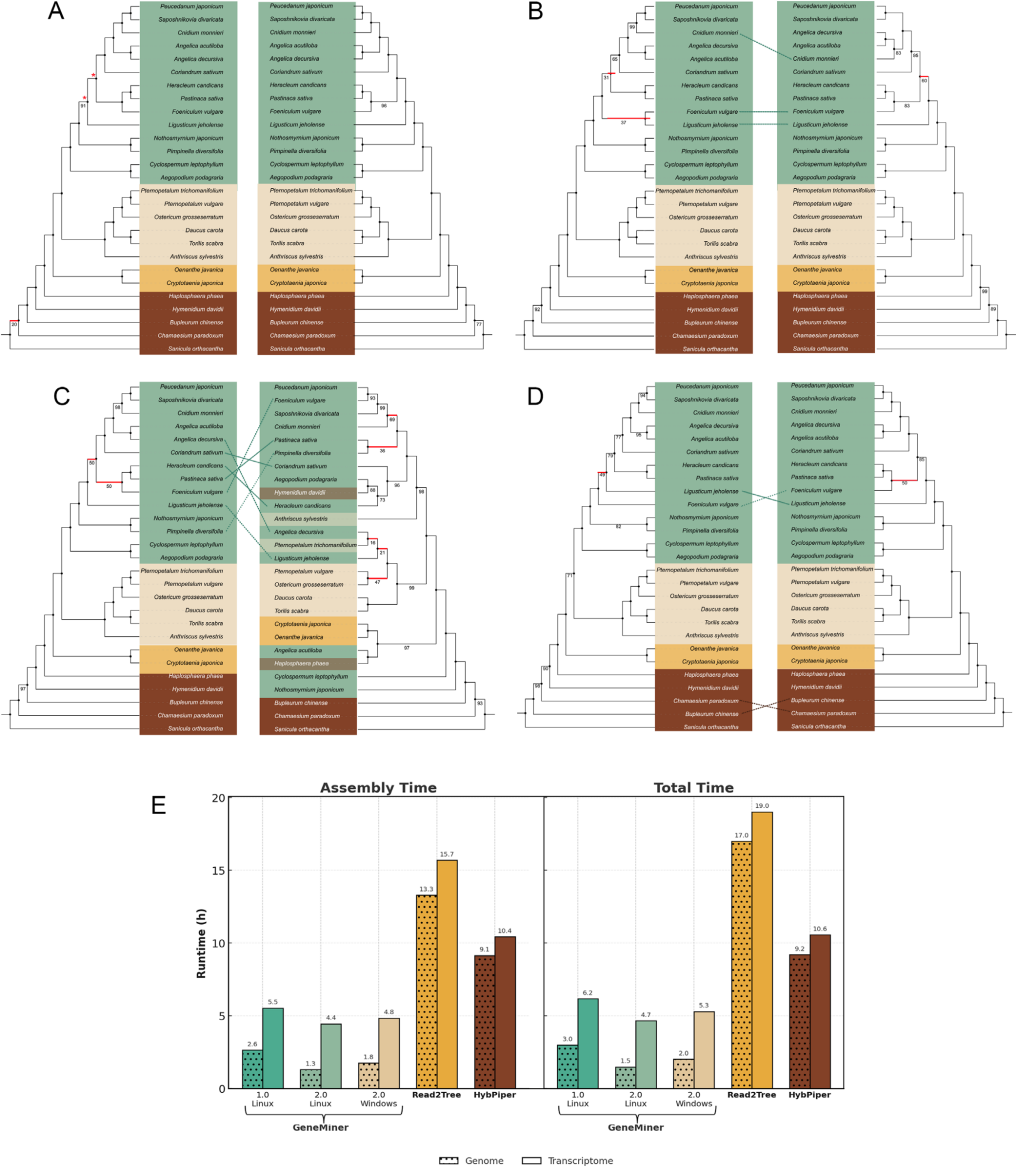
GeneMiner2 produces more stable tree topologies from genome skimming data compared to state‐of‐the‐art pipelines. (A) Phylogenetic trees reconstructed by GeneMiner2 based on SCGs from transcriptome data (left) and genome skimming data (right). Branches with less than 100% support are labelled with their values, while branches with support below 70% are highlighted in red. Large conflicts within groups are indicated with dashed lines. Groups A, B, C and basal branches are represented in green, beige, yellow and brown. Asterisks (*) denote controversial branches indicated in previous research. (B) Phylogenetic trees reconstructed by Read2Tree based on SCGs from transcriptome data (left) and genome skimming data (right). (C) Phylogenetic trees reconstructed from SCGs recovered by HybPiper using the same downstream phylogenetic pipeline, based on transcriptome data (left) and genome‐skimming data (right). (D) Phylogenetic trees reconstructed from SCGs recovered by GeneMiner using the same downstream phylogenetic pipeline, based on transcriptome data (left) and genome‐skimming data (right). (E) Assembly runtimes and entire pipeline runtimes (including assembly, alignment, trimming, and tree reconstruction) for genome skimming data and transcriptome data.

Figure 4 comprises the phylogenetic trees constructed from the single‐copy genes (SCGs) assembled by GeneMiner, GeneMiner2, HybPiper, and Read2Tree. Additional phylogenetic trees are available in the Supporting Information Figures. Benefiting from its accurate assembly algorithm, GeneMiner2 consistently reconstructed highly robust species trees using both transcriptome and genome skimming data (Figures 4A, S1 and S2). Across the inferred phylogenies, all but one of the major clades received bootstrap support values above 70%, with the majority reaching 100%. The phylogenetic tree constructed by GeneMiner2 from genome skimming data and the transcriptome‐based tree showed no topological incongruence, while those reconstructed by GeneMiner exhibited two topological discrepancies, one of which occurred at a basal branch (Figure 4D). For accuracy comparison, we used the tree topology provided by Wen (Wen et al. 2020) as a representative of the state‐of‐the‐art phylogenomic study. Branches with nearly equal quartet support were marked as controversial (Figure 4A). In regions of topological uncertainty, the tree topology recovered by GeneMiner2 was consistent with previous studies and exhibited higher bootstrap support.

The transcriptome‐ and genome‐based trees constructed (Nguyen et al. 2015) under the Read2Tree pipeline exhibited inconsistent topologies and low bootstrap support at controversial branches. Upon inspecting the sequences of taxa involved in controversial branches, we found that these issues were likely caused by the inclusion of paralogous genes. Using the OMA reference set, the trees reconstructed by GeneMiner2 from genome skimming and transcriptome data had identical topologies and showed higher branch support than those reconstructed by Read2Tree (Figure S1).

Under the HybPiper pipeline, the phylogenetic tree inferred from transcriptome‐derived SCG sequences was congruent with that generated by GeneMiner2, although it exhibited slightly lower bootstrap support. The tree constructed from genome skimming data showed evident topological discordance with the transcriptome‐based tree both within and among groups (Figure 4C).

We benchmarked the assembly time and total runtime of the phylogenetic pipeline across different tools (Figure 4E). With the exception of Read2Tree, all pipelines were subjected to a uniform downstream workflow comprising multiple sequence alignment (MAFFT), alignment trimming (trimAL), and phylogenetic inference (FastTree). We observed that GeneMiner2 significantly outperforms Read2Tree and HybPiper in assembly and total runtime. Specifically, on genome skimming data under Linux, GeneMiner2 reduced assembly time by 90.2% compared to Read2Tree (1.30 vs. 13.28 h), and by 85.8% compared to HybPiper (1.30 vs. 9.14 h). For total runtime, it achieved a reduction of 91.3% over Read2Tree (1.48 vs. 16.99 h), and 83.9% over HybPiper (1.48 vs. 9.20 h). On transcriptome data, GeneMiner2 achieved runtime reductions of 71.7% and 57.4% in assembly time, and 77.1% and 66.0% in total time, compared to Read2Tree and HybPiper, respectively. The assembly runtime of GeneMiner2 has been markedly improved compared to the previous version, on both Linux and Windows platforms. Furthermore, the runtime of GeneMiner2 on Windows was only 20–40 min longer than that of Linux, demonstrating strong cross‐platform performance improvements.

## 4 Discussion

### 4.1 Algorithmic Improvements and Enhanced Performance

Our results showed that GeneMiner2 provides a robust and streamlined solution for gene assembly and phylogenetic inference from genome skimming data, even when only distantly related references are available. Building upon the previous version (Xie, Guo, et al. 2024), we optimised three core steps with new algorithms to achieve more accurate and efficient assembly.

An initial improvement involves updating the pre‐assembly filtering step with a more efficient hash table implementation (Figure S13). The optimization of this step greatly improves the speed of GeneMiner2 because the previous version must process all raw reads, perform electronic baiting, and classify each read to specific genes. While modern electronic baiting software often uses minimizers as a sub‐sampling strategy to improve speed, failure to capture certain *k*‐mers can be detrimental to de Bruijn assembly, so we choose not to use any sub‐sampling strategy. Instead, we implemented a specially optimised data structure to allow fast query and iteration. By translating ‘searching for the genes linked to a *k*‐mer’ to exactly one query‐then‐iterate operation, GeneMiner2 greatly outperforms the previous version (Xie, Guo, et al. 2024) and other similar tool implementations. The handling of paired‐end data is also updated in the new version, which salvages short reads if the other read in its pair matches the database.

In addition, the re‐filtering step became more fine‐grained. Increment in *k*‐mer size was previously aggressive and is now more sensitive to coverage. During re‐filtering, a read is first checked whether it can be unambiguously oriented, using a series of statistical tests. This prevents a variety of sequencing artefacts from entering the assembly stage. Furthermore, by excluding some types of inversions, multiple sequence alignment with higher quality can be obtained, ameliorating the accuracy of phylogenetic inference. The statistical tests simply decide if a read contains too many inversions compared to the reference, which is indicative of potential chimerism or degradation. After statistical tests, discordant read pairs are removed. Then the read is filtered with an incrementing *k*‐mer size until a minimum coverage is hit. If the drop in coverage is too sharp, the increment in *k* is reverted to avoid overzealous read removal. We have empirically found that less than 1% of raw reads trigger the statistical tests, suggesting the safety of the re‐filtering strategy. Additionally, the strategy generates an assembly with fewer inversions. Thus, the multiple sequence alignment can generate a supermatrix more accurately, leading to phylogenetic trees that are robust across depths and data types.

Moreover, a module for estimating optimal *k*‐mer size has been incorporated into the assembler, addressing the sensitivity of de Bruijn graph‐based assemblers to *k*‐mer selection. Although several methods exist to estimate *k* for whole genome assemblies, we choose *k* on a per‐gene basis to maximise the extent of sequence recovery. Because regions of a genome can be sequenced at varying depths, it is desirable to estimate *k* without coverage information. For weighted de Bruijn graphs to work, it is necessary for reads and references to share *k*‐mers, prompting the selection of small *k* values. However, small *k* values might cause misassembly. We resolve this by first finding the *k* value with the most shared *k*‐mers and then selecting the largest *k* value with no less than half of the most shared *k*‐mers. Therefore, reads and references will share sufficiently many *k*‐mers without introducing misassembly. The assembled genes can thus have both high contiguity and high quality.

To comprehensively evaluate the assembly performance and phylogenetic reconstruction capabilities of GeneMiner2, simulation experiments were conducted across 1–10× sequencing depths and 0.05–0.2 reference divergences. Benefiting from a redesigned filtering and assembly algorithm, GeneMiner2 substantially improves runtime efficiency while preserving high assembly accuracy. Compared to the conventional assembly and annotation approach, GeneMiner2 substantially reduced the error rates of assembled sequences and improved correctness and completeness under low‐coverage conditions. It successfully assembled target genes under low‐coverage conditions, with over 80% of the sequences being error‐free, and maintains robustness and accuracy even with distantly related references (up to 0.2 divergence).

### 4.2 Applications and Future Directions

Advances in short read sequencing technologies and the continuous decline in sequencing costs have made large‐scale genome skimming feasible. Conventional genome assembly methods typically require high‐coverage DNA sequencing libraries and reference genomes, along with computationally intensive reconstruction processes. Indeed, genome skimming data are sufficient for assembling low‐copy genes. Targeted approaches such as Read2Tree employ mapping‐based strategies that bypass complete genome assembly, enabling analysis directly from genome skimming data. However, these approaches still rely heavily on high‐quality orthologous gene databases. Although the continued expansion of large‐scale orthologous databases such as OMA (Altenhoff et al. 2019), OrthoDB (Kuznetsov et al. 2023), and EggNOG (Huerta‐Cepas et al. 2019) has partially alleviated this issue, database development for low‐level taxa remains incomplete.

GeneMiner2 offers an effective solution for low‐level taxa lacking high‐quality reference, leveraging an efficient assembly algorithm that enables reconstruction using only a limited set of orthologous groups derived from transcriptomes. In this paper, we retrieved orthologous groups from Apiaceae transcriptomes as references and assembled over 2000 SCGs based on genome skimming data. GeneMiner2 outperformed other pipelines in both assembly time and completeness on the Apiaceae dataset. It recovered more genes and produced fully congruent phylogenies with strong bootstrap support across both transcriptome and genome skimming data, unlike HybPiper and Read2Tree, which showed limited recovery or topological inconsistencies.

Despite advances, GeneMiner2 has its theoretical and practical limitations, as targeted gene assemblers generally exhibit limited performance in assembling introns. When reference sequences are derived from transcriptome data, accurately identifying intron‐containing reads from genomic data remains a significant challenge. Future versions of GeneMiner may incorporate specialised mapping algorithms alongside AI‐based methods to improve intron assembly, enabling precise whole‐gene reconstruction directly from raw reads and potentially overcoming current theoretical limitations.

GeneMiner2 is a multifunctional and efficient tool designed for target gene assembly from genome skimming data. It offers streamlined workflows for raw reads assembly, ortholog identification, phylogenetic reconstruction and divergence time calibration, all executable through a user‐friendly graphical interface. The tool can serve a broad community of evolutionary biologists applying large scale DNA sequence data for evolutionary analyses, including, but not limited to, researchers interested in organismal phylogeny, lineage tracing of cells in cancer phylogenetics.

## Author Contributions

Y.Y. and X.Y. conceived and designed the study. Y.Y. and Z.T. implemented the software. X.Y. and Z.T. performed data analysis, conducted code review, and drafted the initial manuscript. Z.Z., Y.S., H.H., Y.S., and J.H. provided feedback on software functionality and assisted in proofreading. All authors contributed to editing and approved the final manuscript.

## Funding

This work was funded by the Science & Technology Fundamental Resources Investigation Program (grant no. 2022FY101000), the National Natural Science Foundation of China (grant nos. 32271552 and 32571738), and supported by Sichuan Natural Science Foundation (grant no. 2026NSFSCZY0116).

## Conflicts of Interest

The authors declare no conflicts of interest.

## Supporting information


**Data S1:** men70111‐sup‐0001‐DataS1.docx.

## Data Availability

Supporting Information, scripts, simulated sequencing data and reference datasets are available at https://github.com/yxyhahaha/paper_work. The source code for GeneMiner2 is available at https://github.com/sculab/GeneMiner2. The packaged executable tool is available for download at https://sourceforge.net/projects/geneminer.
