# Supplementary material for: GeneMiner2: Accurate and Automated Recovery of Genes From Genome Skimming Data
Source: Mol Ecol Resour. 2026 Feb 18;26(2):e70111. doi: 10.1111/1755-0998.70111 (PMC12914762; doi:10.1111/1755-0998.70111)
Supplement: Supplementary file 1 — Data S1: men70111‐sup‐0001‐DataS1.docx. [file MEN-26-e70111-s001.docx]

## Supporting Information

**
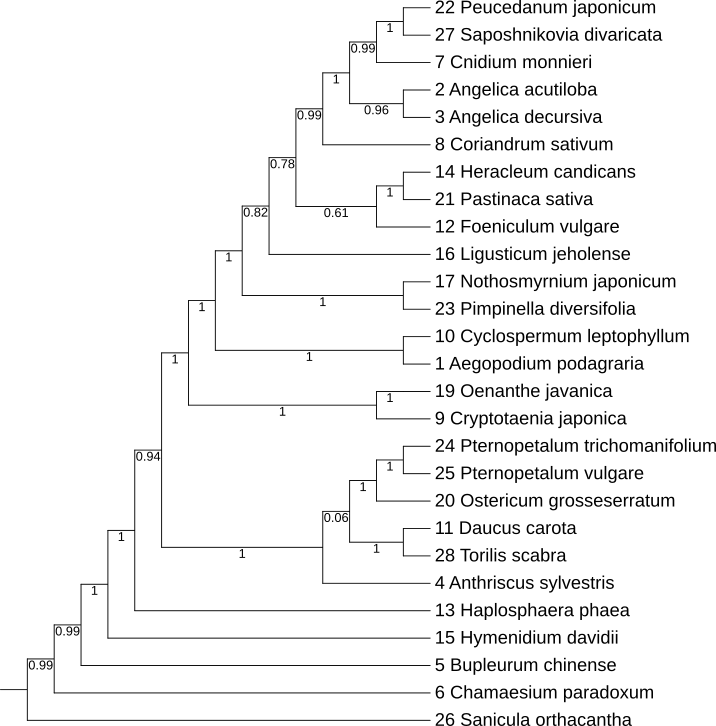
**

**Figure S1.** The concatenated tree constructed by GeneMiner2 from genome skimming data (using the OMA database as reference), no topological conflict.

**
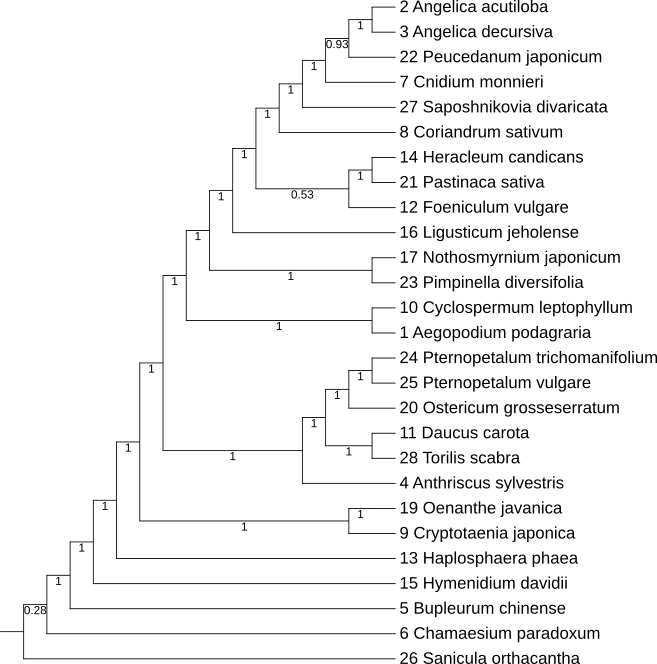
**

**Figure S2.** The concatenated tree generated by GeneMiner2 from genome skimming data (using 353 genes as reference) exhibits one topological conflict.


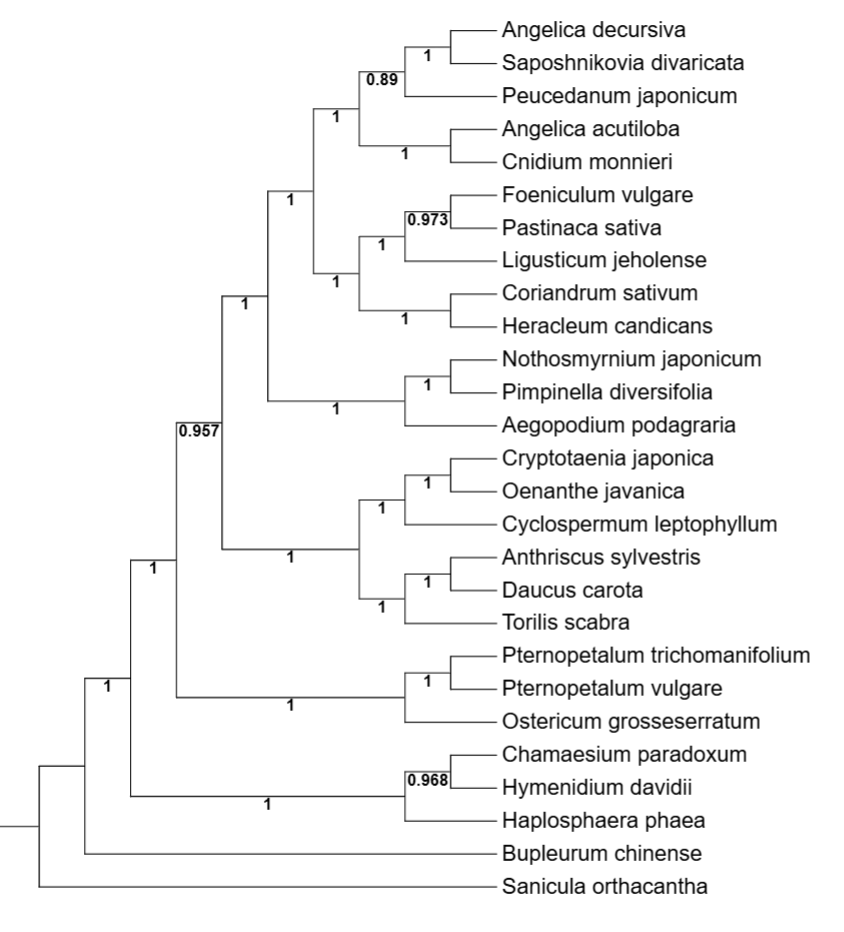
**Figure S3.** The concatenated tree generated by GeneMiner from genome skimming data (using 353 genes as reference) exhibits one topological conflict.


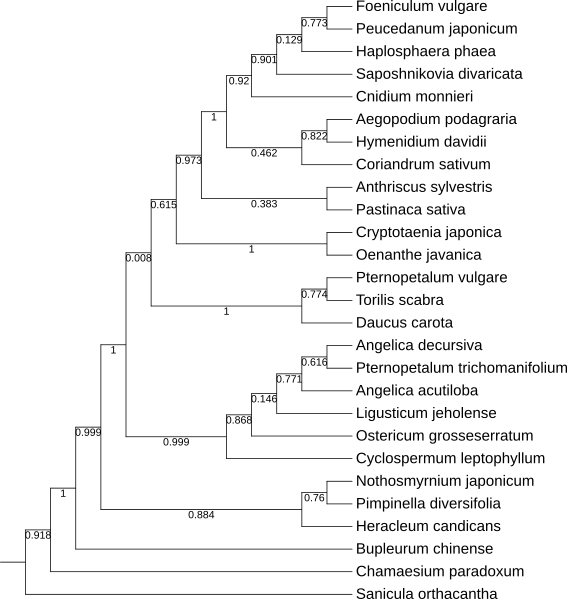


**Figure S4.** The concatenated tree generated by HybPiper from genome skimming data (using 353 genes as reference) after paralog filtering. The tree shows extremely low support at certain nodes, with the minimum value reaching only 0.008.


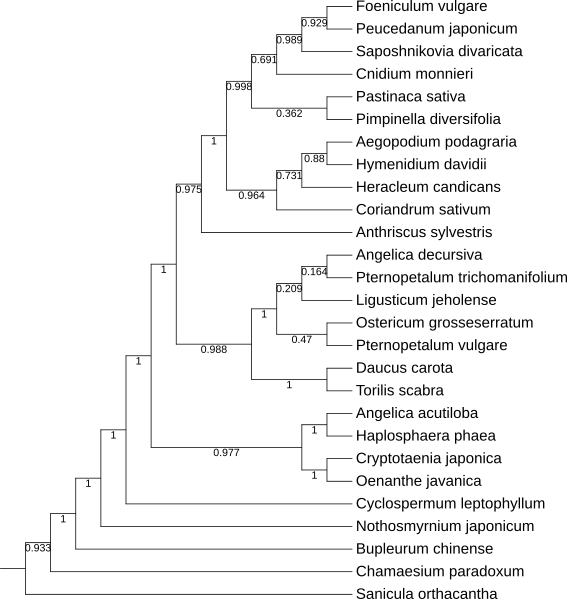


**Figure S5.** The concatenated tree generated by HybPiper from genome skimming data (using 353 genes as reference) without paralog filtering.


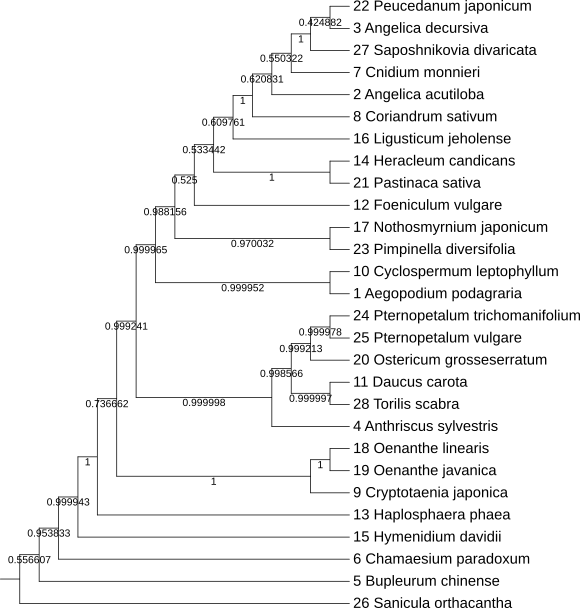


**Figure S6.** The coalescent-based tree generated by GeneMiner2 from genome skimming data (using 353 genes as reference) .


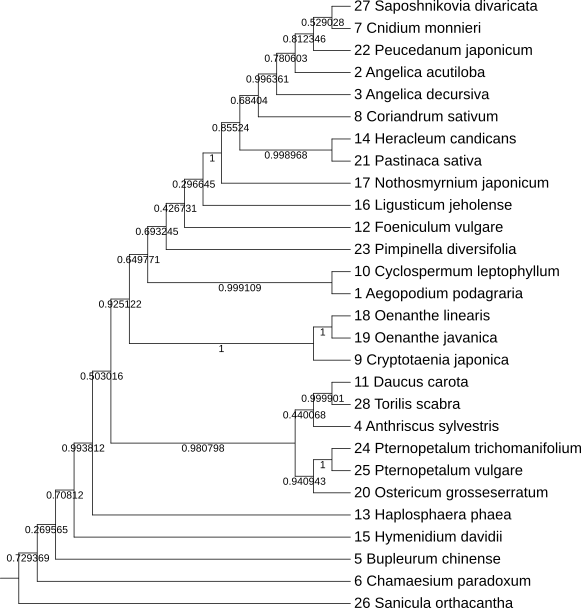


**Figure S7.** The coalescent-based tree generated by GeneMiner2 from genome skimming data (using the OMA database as reference) .


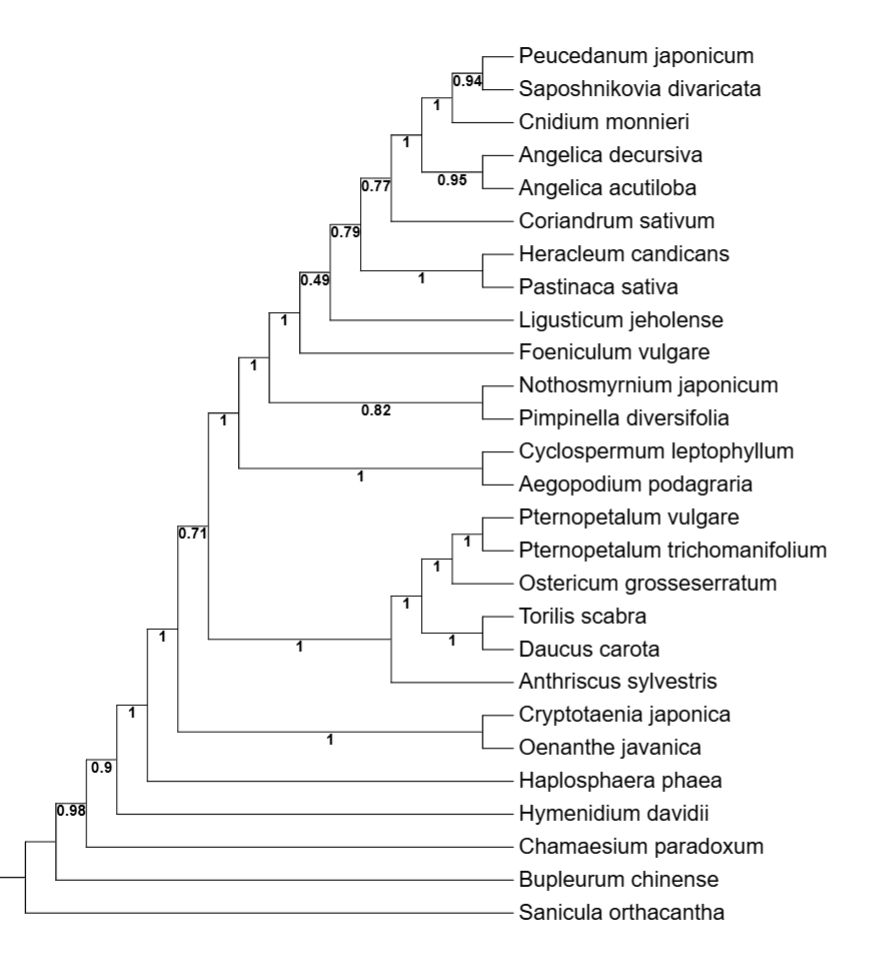


**Figure S8.** The coalescent-based tree generated by GeneMiner from genome skimming data (SCGs database as reference) .


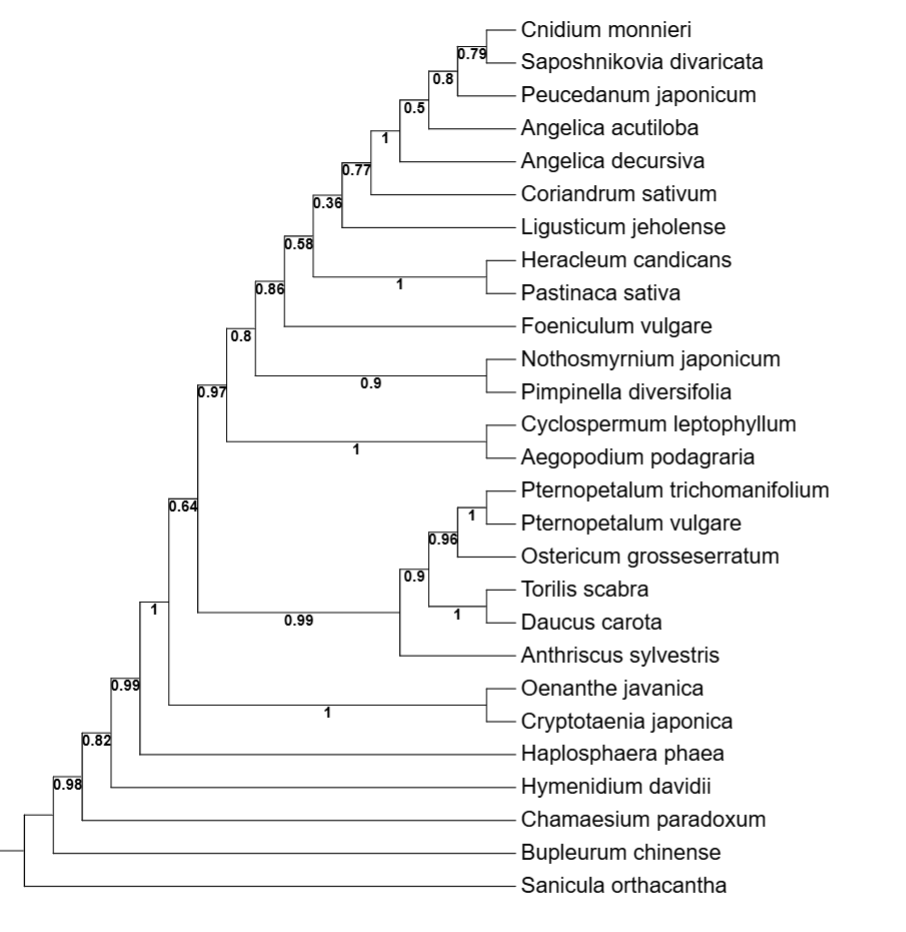


**Figure S9.** The coalescent-based tree generated by GeneMiner from genome skimming data (353 genes database as reference) .


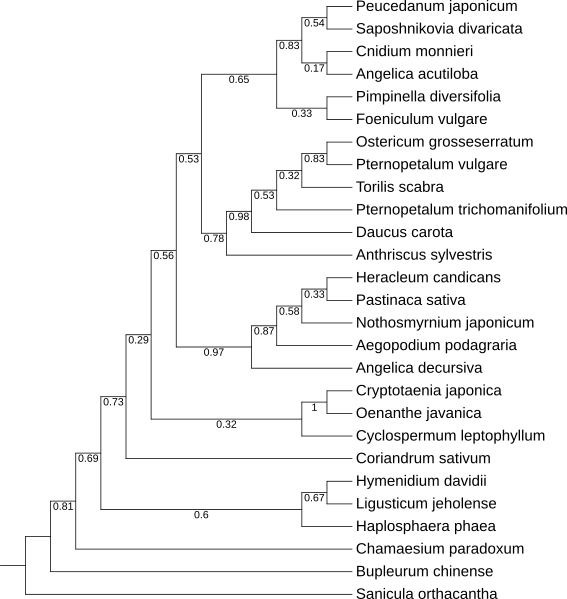


**Figure S10.** The coalescent-based tree generated by HybPiper from genome skimming data (using 353 genes as reference) without paralog filtering.


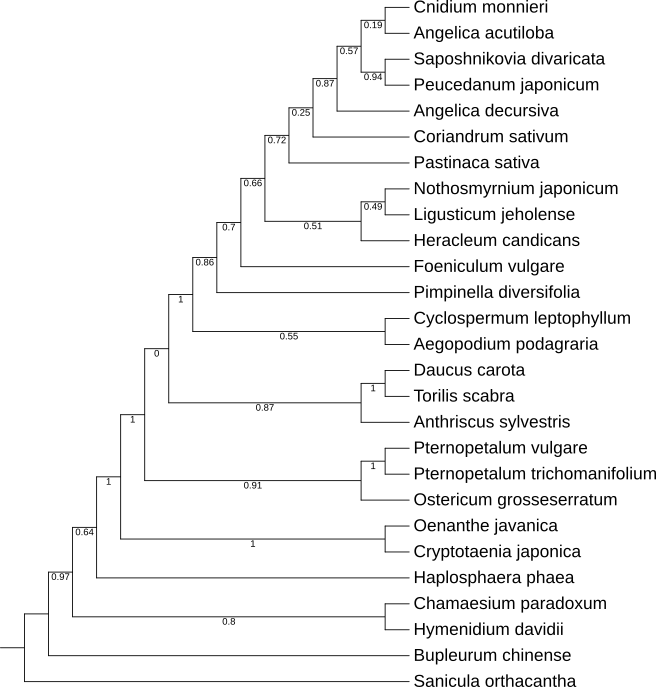


**Figure S11.** The coalescent-based tree generated by HybPiper from genome skimming data (using 353 genes as reference) after paralog filtering. HybPiper failed to assemble a sufficient number of SCGs to construct a concatenated tree.


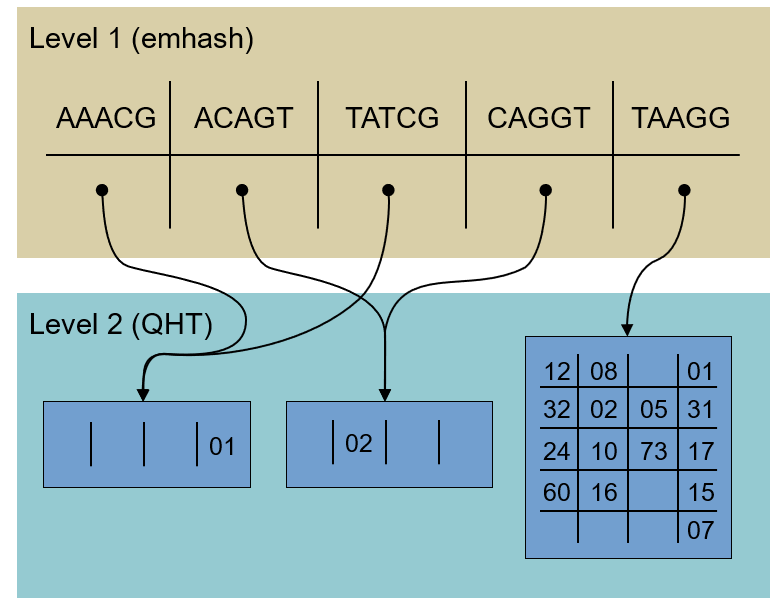


**Figure S12.** Illustration of the organization of the *k*-mer database in GeneMiner2. The Level 1 hash table (emhash) stores k-mers as keys and maps them to corresponding entries in Level 2 (QHT), which compactly stores gene indices. This structure enables fast query and iteration without subsampling, avoiding false negatives in de Bruijn assembly. The design combines efficient memory usage with high lookup speed, supporting reliable k-mer-to-gene mapping in large-scale data.


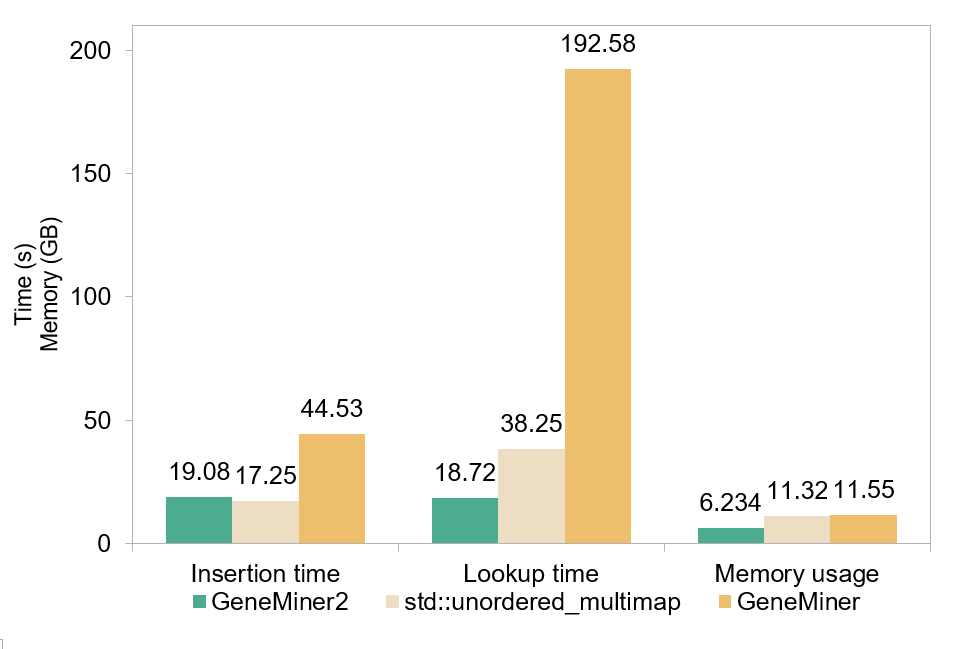
**F****igure S13.** The time taken and the memory consumed to insert 50 million *k*-mers and look up 1 billion *k*-mers in each multimap implementation. Each *k*-mer is randomly associated with 10 out of 1,000 genes. For std::unordered_multimap in the C++ standard library, we used the implementation shipped with GCC 14. All benchmarks are single-threaded.

*variable region*

*reference*

*true sequence*

*assembly result*

**Figure S14.** A case where an inverted chimera causes a misassembly. The orange segment represents a read which can be mapped to the reference from two directions. The red, yellow and purple dots indicate mismatched bases between reads and reference. As the orange chimera matches the reference with fewer errors, it is assigned a higher weight than the green, structurally correct reads. Palindromic misassembly ensues when assembly progresses past the chimeric junction (dotted orange line) and continues in the reverse direction.


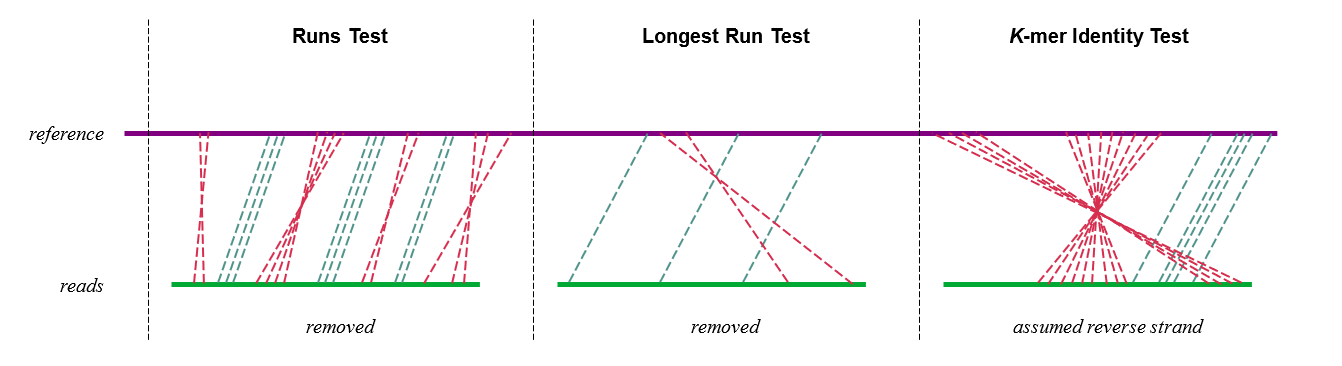


**Figure S15.** Illustration of the statistical tests employed in read filtering. Reads with frequent inversions are removed using the runs test. Reads without multiple consecutive *k*-mer hits are removed using the longest run test. Reads that seem to span an inversion are checked further; only when the hits in two directions have different similarity to the reference will the read be kept.

**Table S1.** The mean assembled sequence length, the mean number of sequences per gene file (species count), and the number of assembled sequences (seq count) across different tools based on the genomic dataset, in the empirical data test.

| **Tools** | **353 genes**  **/length** | **353 genes**  **/species count** | **SCGs**  **/length** | **SCGs**  **/species count** | **SCGs**  **/seq count** | **SCGs refs**  **/seq count** |
| --- | --- | --- | --- | --- | --- | --- |
| GeneMiner2.0 | 243.8642 | 26.912 | 241.5167 | 20.6969 | 2201 | 2605 |
| GeneMiner1.0 | 378.9231 | 26.7337 | 314.7034 | 18.9317 | 2021 |  |
| HybPiper | 455.7455 | 6.64 | 479.3464 | 5.27 | 2033 |  |
| Read2Tree | - | - | 1141.423 | 15.89 | 200 | 200 |

**Table S2.** The number of single-copy genes (strictly single-copy only) extracted by GeneMiner2 via OrthoFinder from transcriptome data.

| **Number of species \blastn** | **1.00E-50** | **1.00E-20** | **0.01** | **0.1** |
| --- | --- | --- | --- | --- |
| 0 | 6743 | 6485 | 6485 | 6485 |
| 1 | 6743 | 6485 | 6485 | 6485 |
| 2 | 6743 | 6485 | 6485 | 6485 |
| 3 | 2605 | 2553 | 2552 | 2552 |
| 4 | 1279 | 1248 | 1245 | 1245 |
| 5 | 694 | 675 | 669 | 669 |
| 6 | 385 | 368 | 359 | 359 |
| 7 | 218 | 209 | 211 | 211 |
| 8 | 135 | 129 | 128 | 128 |
| 9 | 86 | 81 | 82 | 82 |
| 10 | 52 | 48 | 47 | 47 |
| 11 | 36 | 32 | 30 | 30 |
| 12 | 21 | 19 | 18 | 18 |
| 13 | 13 | 12 | 12 | 12 |
| 14 | 10 | 10 | 10 | 10 |
| 15 | 9 | 9 | 9 | 9 |
| 16 | 6 | 6 | 6 | 6 |
| 17 | 5 | 5 | 5 | 5 |
| 18 | 4 | 4 | 4 | 4 |
| 19 | 3 | 3 | 3 | 3 |
| 20 | 0 | 1 | 1 | 1 |
| 21 | 0 | 1 | 1 | 1 |
| 22 | 0 | 0 | 0 | 0 |
| 23 | 0 | 0 | 0 | 0 |
| 24 | 0 | 0 | 0 | 0 |
| 25 | 0 | 0 | 0 | 0 |
| 26 | 0 | 0 | 0 | 0 |
| 27 | 0 | 0 | 0 | 0 |
| 28 | 0 | 0 | 0 | 0 |

**Table S3.** Numbers of recovered sequences (L1, L2) at a reference sequence divergence of 0.1, with high identity (identity = 100%) and high recovery (7–10 recovered sequences).

| Seq_depth | 1× | 2× | 3× | 4× | 5× | 6× | 7× | 8× | 9× | 10× |
| --- | --- | --- | --- | --- | --- | --- | --- | --- | --- | --- |
| GeneMiner1.0 | 109 | 463 | 661 | 741 | 739 | 740 | 737 | 729 | 728 | 759 |
| GeneMiner2.0 | 879 | 869 | 862 | 862 | 833 | 817 | 824 | 780 | 792 | 769 |
| The  conventional  approach | 0 | 1 | 14 | 10 | 16 | 23 | 64 | 70 | 146 | 203 |

**Table S4.** FN (false negative) values in the simulated data test at a reference sequence divergence of 0.1, obtained by comparing trees reconstructed using the concatenation-based method with the standard tree.

| Seq_depth | 1× | 2× | 3× | 4× | 5× | 6× | 7× | 8× | 9× | 10× |
| --- | --- | --- | --- | --- | --- | --- | --- | --- | --- | --- |
| GeneMiner1.0 | 4 | 2 | 5 | 2 | 3 | 2 | 1 | 1 | 0 | 1 |
| GeneMiner2.0 | 2 | 0 | 0 | 0 | 0 | 0 | 0 | 0 | 0 | 0 |
| The  conventional  approach | 7 | 6 | 3 | 3 | 2 | 4 | 0 | 0 | 1 | 0 |

**Table S5.** FN (false negative) values in the simulated data test at a reference sequence divergence of 0.1, obtained by comparing trees reconstructed using the coalescent-based method with the standard tree.

| Seq_depth | 1× | 2× | 3× | 4× | 5× | 6× | 7× | 8× | 9× | 10× |
| --- | --- | --- | --- | --- | --- | --- | --- | --- | --- | --- |
| GeneMiner1.0 | 1 | 0 | 1 | 0 | 0 | 1 | 1 | 1 | 1 | 0 |
| GeneMiner2.0 | 1 | 0 | 0 | 0 | 0 | 0 | 0 | 0 | 0 | 0 |
| The  conventional  approach | 6 | 5 | 0 | 0 | 0 | 0 | 0 | 5 | 0 | 0 |
